# Enhancement of thermal performance in rectangular solar air heater duct using tetrahedron-shaped turbulators

**DOI:** 10.1038/s41598-025-27762-8

**Published:** 2025-11-28

**Authors:** Anoop Kanjirakat, Anuraag M. Kamath, Sohail A. Khan, V. Shubhamkar, Dolfred Vijay Fernandes

**Affiliations:** 1 Department of Aeronautical and Automobile Engineering, Manipal Institute of Technology, Manipal Academy of Higher Education, Manipal, Karnataka 576104 India; 2 Department of Mechanical and Industrial Engineering, Manipal Institute of Technology, Manipal Academy of Higher Education, Manipal, Karnataka 576104 India

**Keywords:** Heat exchanger, CFD, Vortex generator, Thermo-hydraulic performance, Heat transfer enhancement, Friction factor, Devices for energy harvesting, Solar thermal energy, Mechanical engineering, Fluid dynamics, Computational science

## Abstract

This study investigates the effectiveness of tetrahedron-shaped turbulators attached to the bottom surface of a heated absorber plate to increase heat transfer in solar air heaters (SAHs). By utilizing three-dimensional computational fluid dynamics analysis, this study evaluates the impact of these turbulators on the heat transfer while accounting for friction losses in an in-line arrangement. The tetrahedron geometry significantly improves the heat transfer efficiency, with the thermo-hydraulic performance (THP) parameter serving as a quantitative metric for assessing heat transfer enhancement, incorporating friction energy losses and aiding in the optimization of turbulator geometry. The results of a parametric study indicate that a reduced relative longitudinal pitch (*P*/*D*) ratio significantly enhances the effectiveness of turbulators at a constant Reynolds number. Additionally, increased turbulator height correlates with improvements in the Nusselt number by facilitating better mixing within the SAH. The leading-edge angle of the turbulator emerges as a critical determinant of THP, with a negative angle $\left(\beta =-30^\circ \right)$ demonstrating significant performance enhancements over a neutral angle $\left(\beta =0^\circ \right)$, which is attributed to improved mixing dynamics and reduced frictional resistance. The study identifies an optimal THP value of 1.78, which is achieved with $(P/D)=0.812$,$(e/D)=0.27$,$(p/D)=0.75$, and $\beta =-30^\circ$ at a Reynolds number of 9000.

## Introduction

Renewable solar power sources remain largely underutilized, primarily due to the low efficiency of systems that convert solar energy into usable work. Nevertheless, efforts are continuously being made to increase the efficiency of these systems through various design changes. Solar air heaters (SAHs) are systems that harness sunlight and facilitate the warming of the air that flows through them^1^. These systems include a collector that absorbs solar radiation and converts it into thermal energy. As air passes through the duct located beneath the absorber or collector plate, its temperature increases. This warm air is then used for various applications, such as heating spaces or drying materials^2,3^. SAHs are eco-friendly and budget-friendly and require minimal maintenance, making them ideal for homes, businesses, and industrial settings^4^.

Solar air heaters often incorporate flat plate absorbers because of their simplicity in design, ease of application, and minimal maintenance requirements. In addition to the fact that air has low specific heat values, the heat transfer coefficient in smooth channels is also low, primarily because of the substantial hydrodynamic boundary layer. To improve heat transfer from the heating surface, various modifications to the heated absorber plate surface can be utilized to effectively disrupt the boundary layer. Among the proposed enhancements for increasing the heat transfer coefficient between the heating surface and the fluid are the addition of fins to the heating surface, baffles with fins, lateral ribs, turbulators, and vortex generators. Although these modifications have been shown to significantly improve the thermal efficiency, they also lead to an increased pressure drop at higher fluid flow rates^5^. In addition to these modifications, various flow geometries, such as triangular, trapezoidal, and semicircular shapes, for SAHs have also been explored in the past^6–9^.

Among the various techniques used to increase the thermal performance of SAHs, the use of turbulators or turbulence promoters seems promising^10–12^. The turbulators act passively to increase heat transfer without increasing the overall surface area. By promoting flow mixing and disrupting the boundary layer on the heated absorber plate surface, these turbulators ultimately improve the thermal efficiency of SAH. The effect of turbulators in improving heat transfer has been studied experimentally and numerically in the past. Computational fluid dynamics (CFD) offers a cost-effective method of analysis to study the impact of tabulators on the flow and thermal characteristics of air within a solar air heater^13^. Since the use of turbulators also increases the pressure drop, especially at higher Reynolds numbers, the improvement in heat transfer needs to be analyzed along with the penalty for pressure drop. The THP parameter, which is the ratio of thermal enhancement to pressure drop increase, is an ideal metric for comparing various tabulators^14^.

Turbulators can be broadly classified into rib types (rectangular, circular, triangular, NACA profiles, V-shaped) and vortex generator types (cylinders, cones, springs, winglets), which generate strong lateral and longitudinal vortices to increase heat transfer rates within SAHs^15–18^. Chaube et al.^19^ conducted a 2D numerical study of solar air heaters with ribs of different shapes (square, rectangular, chamfered, circular, semicircular, and triangular) and reported that rectangular ribs have better thermo-hydraulic performance. Kumar et al.^20^ performed 2D-CFD analysis of symmetric half NACA 0020-shaped ribs in both forward and reverse orientations. A maximum THP value of 2.2 (at $Re=6000$) was observed when the rib was used in the reverse orientation. The numerical observations were also compared with the experimental data. Singh et al.^21^ both experimentally and numerically examined the performance of down-configured quarter-circular and half-trapezoidal ribs in a curved SAH. Compared with the half trapezoidal ribs, the down-configured quarter-circular ribs resulted in a 10–12% increase in the THP. Yadav and Bhagoria^22^ studied the thermal performance of equilateral triangular ribs on an absorber plate. The THP parameter of 2.11 (at $Re=15000$, $P/e=7.14$ and $e/D=0.042$, where $P$ is the longitudinal distance between the turbulators, $e$ is the turbulator height, and $D$ is the hydraulic diameter) was observed via 2D flow analysis. Gawande et al.^23^ experimentally and numerically studied the thermal performance of reverse L-shaped ribs on absorber plates. They observed a peak THP value of 1.90 at $Re=15000$ ($P/e = 7.14$ and $e/D = 0.042$). The studies mentioned above were conducted via 2D-CFD simulations. While these simulations effectively predict the longitudinal variation in flow characteristics required for rib-type turbulators, they fall short in terms of analyzing lateral flow effects. In such cases, 3D simulations are necessary, as the vortices formed will also occur in the transverse directions.

Kumar and Kim^24^ analyzed V-shaped ribs in solar air heaters in the 3D-CFD domain. Multiple ribs (in the form of thin wires) were patterned in V shape with gaps between them. The optimum thermal hydraulic performance parameter, with a peak value of 3.6, was achieved with a roughness geometry corresponding to a gap-to-rib height ratio of 1.0. For similar V-patterned ribs, Sharma and Bhargva^25^ reported an effective efficiency of 74% (at $Re=\text{14,000}$) for a relative roughness height of 0.034. Pandey et al.^26^ investigated the effects of 45° V-down baffle blocks with staggered racetrack-shaped perforation geometries on SAHs in an experimental study. The study revealed that the use of a baffle block with an $e/D$ ratio of 0.7 and a $P/e$ value of 2 led to an increase of 456% in the Nusselt number and a 39-fold increase in the friction factor compared with those of a smooth duct. A maximum THP value of 1.43 was observed experimentally.

The flow effects caused by vortex generator-type turbulators are studied via 3D-CFD simulations to capture the lateral flow effects. Alam and Kim^27^ numerically investigated the effect of conical protrusions on the absorber plate of a solar air heater. The maximum enhancements recorded are 2.49 for the Nusselt number and 7.29 for the friction factor. These improvements occurred at $P/e=10$ and $e/D=0.044$. Antony et al.^28^ evaluated the performance of stepped cylindrical protrusions, with each turbulator having 1 to 3 steps. The core diameter and relative roughness pitch ratio ranged from 3 to 7 mm and 11 to 27, respectively. An increase in the number of steps significantly elevated the localized Nusselt number over the turbulators. This enhancement can be attributed to the formation of a vortex flow, which improves the thermal performance. In the case of a three-row configuration, a maximum THP value of 1.49 was recorded at a Reynolds number of 18,000. Bezbaruah et al.^29^ studied the thermohydraulic performance of truncated half-conical vortex generators on an SAH. Air was directed onto the turbulators at different angles of attack. The 60° angle created more powerful vortices with a well-defined core, which significantly disrupted airflow across the transverse plane and improved the thermal performance. A maximum THP value of 2.09 was observed, with a peak thermal enhancement factor of 3.11. Turbulators in the shape of spheres were studied by Manjunath et al.^30^. This study examined the thermal performance of spheres with diameters between 5 and 25 mm, considering relative roughness pitches ($P/D$) of 3, 6, and 12. Compared with the base model, the Nusselt number increased by a factor of 2.52 (at $Re=23560$ and a relative roughness pitch ($P/D$) of 3). Arun Kumar et al.^31^ investigated the effect of a spring-shaped turbulator attached to the bottom of the absorber plate. The THP parameter reached a value of 1.268 for a helicoidal spring with a diameter ratio of 0.06, particularly at lower Reynolds numbers. Similar observations were made by Purohit^32^ in an earlier study with a helicoidal spring turbulator.

Winglet-type vortex generators are widely utilized in SAHs because of their ease of manufacturing and implementation^33^. The perforated winglet-type vortex generators on the absorber were experimentally and numerically studied by Skullong et al.^34^. To minimize pressure loss, holes were punched into the rectangular and trapezoidal winglets, which ultimately enhanced the thermal performance compared with that of the solid winglets. Provonge et al.^35^ studied punched and flapped delta winglet usage in SAHs. The flapped delta winglet was created by partially covering the punched hole with a circular flap, resulting in a 2.9% performance improvement and a THP of 2.16.

As seen from the review of the literature, various kinds of turbulator geometries have been studied to enhance the thermal performance of a solar air heater. The complex, multifaceted geometries induce strong flow disturbances; however, they fail to achieve a better trade-off between flow disruption and pressure drop. Therefore, a simpler hydrodynamic geometry inducing a lesser pressure drop and moderate flow disturbances is considered in the study. In addition, it is hypothesized that tetrahedral geometry induces longitudinal vortices, and the vortex trapping will be minimal due to the presence of an upper sloping surface. Further, to the best of the author’s knowledge, a tetrahedral-shaped vortex generator has not been studied. Hence, in this work, we explore how tetrahedral turbulators enhance heat transfer from the heated absorber plate of a solar air heater. The tetrahedral turbulators are attached to the bottom surface of the heated absorber plate in an inline configuration. A numerical analysis of three-dimensional fluid flow and heat transfer in a solar air heater is conducted. The effects of the longitudinal pitch, turbulator height, and leading-edge angle of the turbulator are studied for various air flow rates in the turbulent flow regime through a SAH. To ensure consistent conditions, the relative lateral pitch ($p/D$) and the angle of attack ($\alpha$) were respectively maintained at 0.750 and 30⁰ throughout the tests. The values of these parameters were fixed based on the existing literature^30,34,36,37^ and the geometric constraints imposed by the absorber plate size.

## Numerical methodology

### Computational domain

**Fig. 1:**
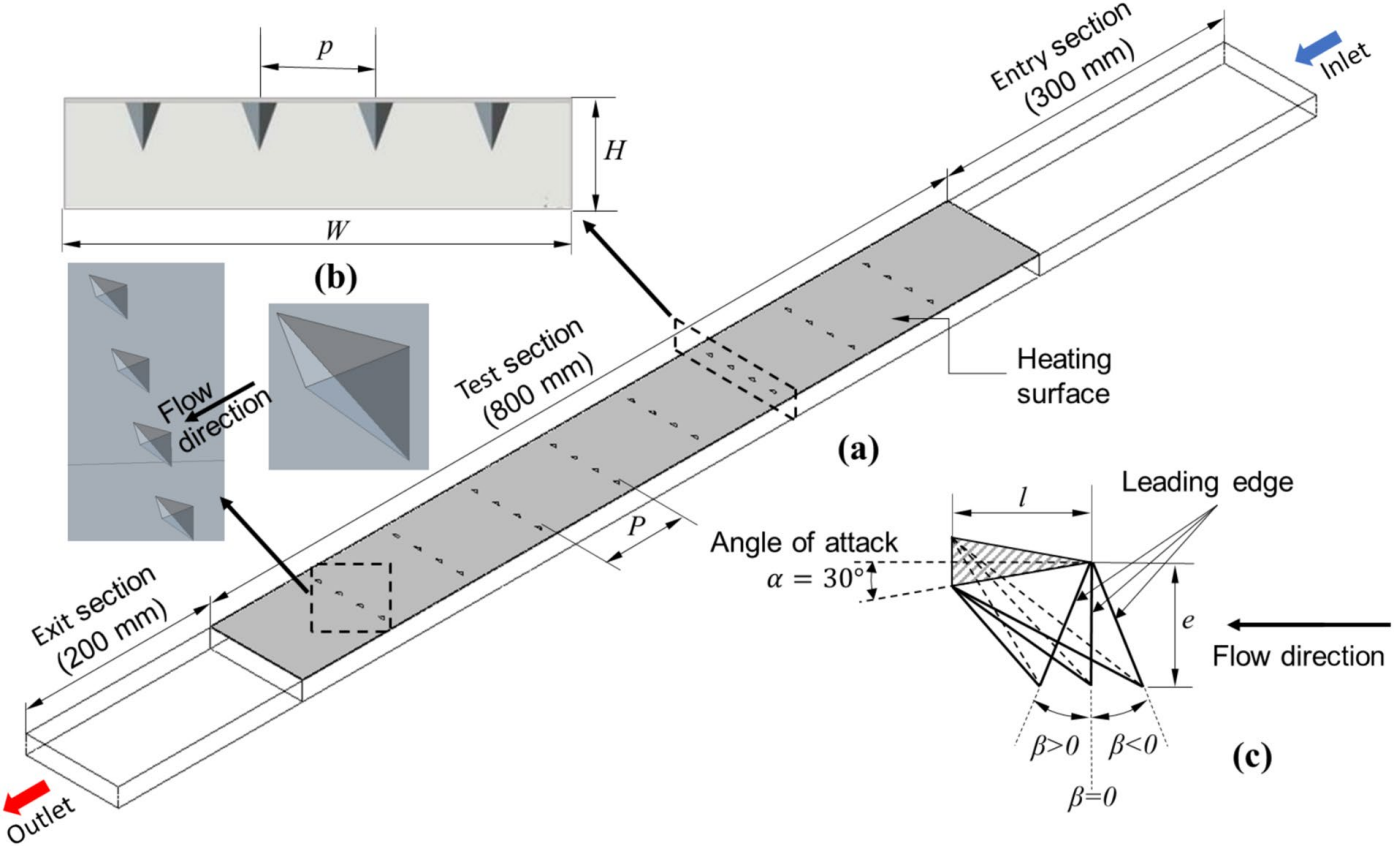
SAH with tetrahedron turbulator roughness on the bottom side of the heated surface: (**a**) computational domain, (**b**) duct sectional view and 3D view of turbulator, and (**c**) geometric details of the tetrahedron turbulators.

The SAH of the present study is a rectangular duct that consists of a heated absorber plate on the upper side, and the remaining three surfaces are adiabatic (insulated). Figure 1a shows the computational domain with the tetrahedron-shaped turbulators attached to the bottom side of the heated absorber plate. The computational domain is divided into three sections: entry, test, and exit. The length of the test section is 800 mm. To ensure complete flow development, an extension is provided at the entrance, with a length of 300 mm as the entry section, and to achieve a uniform temperature of the exiting air stream, a mixing length of 200 mm is provided as the exit section. The values of entry and exit lengths are greater than $5\sqrt{W\times H}$ and $2.5\sqrt{W\times H}$ , respectively, are taken as per the ASHRAE standards^38^. The cross-section $\left(W\times H\right)$ of the duct is 100 mm × 20 mm with a hydraulic diameter $\left(D=2\left(W\times H\right)/\left(W+H\right)\right)$ of 33.33 mm.

The smooth heated surface of the duct is roughened by attaching tetrahedron turbulators intermittently, as shown in Fig. 1a. One of the triangular faces of the tetrahedron is attached to the heated surface (hatched surface in Fig. 1c), whereas the remaining 3 triangular faces are exposed to the air that flows through the duct. This increases the heat transfer area to some extent. However, the primary enhancement in heat transfer is expected to be due to the disruption of the laminar sublayer and the increased turbulence created by the presence of turbulators. The size and arrangement of these turbulators significantly influence the extent of the improvement in heat transfer. The details of the turbulator geometry considered in the study can be seen in Fig. 1c. In all the configurations, the turbulators are arranged in four columns and multiple rows. For example, the 32 turbulators in the $P/D=2.55$ configuration are arranged in four columns and eight rows. Along with the flow Reynolds number, the effects of the turbulator height, longitudinal pitch, and leading-edge angle are studied for fixed angle of attack (30°) and unit roughness height-to-base length ratio ($e/l=1.0$). The angle of attack refers to the angle between the flow direction and the surface of the turbulator on which air impinges. Table 1 presents the range of dimensionless (scaled according to the hydraulic diameter of the duct) geometric and placement parameters used in the study.

**Table 1:** Turbulator geometry and arrangement parameter ranges used in the CFD analysis.

Parameter →	Reynolds no	Relative longitudinal pitch		Relative height		Leading edge angle	Relative lateral pitch	Angle of attack
Study ↓	Re	P/D	No. of turbulators	e/D	e (mm)	β (deg)	p/D	α (deg)
Smooth	9000–24,000	–	–	–	–	–	–	–
Effect of pitch	9000–24,000	2.550	32	0.09	3	0	0.75	30
		1.116	68					
		0.812	92					
		0.450	164					
Effect of height	9000–24,000	0.812	92	0.09	3	0	0.75	30
				0.15	5			
				0.21	7			
				0.27	9			
Effect of turbulator angle	9000–24,000	0.812	92	0.27	9	− 45 to 45	0.75	30

### Numerical model and boundary conditions

The fluid flow and associated heat transfer are governed by the continuity, momentum, and energy equations shown in Eqs. (1), (2), and (3), respectively.

1 $$\frac{\partial }{{\partial x_{i} }}\left( {\rho u_{i} } \right) = 0$$

2 $$\frac{\partial }{{\partial x_{i} }}\left( {\rho u_{i} u_{j} } \right) = \frac{\partial p}{{\partial x_{j} }} + \frac{\partial }{{\partial x_{i} }}\left[ {\mu_{eff} \left( {\frac{{\partial u_{i} }}{{\partial x_{j} }} + \frac{{\partial u_{j} }}{{\partial x_{i} }}} \right)} \right] + \frac{\partial }{{\partial x_{i} }}\left( { - \overline{{\rho u_{i}^{\prime } u_{j}^{\prime } }} } \right)$$

3 $$\frac{\partial }{{\partial x_{i} }}\left( {\rho u_{i} T} \right) = \frac{\partial }{{\partial x_{j} }}\left[ {\left( {\Gamma + \Gamma_{t} } \right)\frac{\partial T}{{\partial x_{j} }}} \right]$$

where $u_{i}$ represents the velocity vector, $p$ represents the pressure, $T$ represents the temperature and $\overline{{\rho u_{i}^{\prime } u_{j}^{\prime } }}$ represents the Reynolds stress tensor. The fluid property density, viscosity, and thermal diffusivity are denoted by $\rho ,\,\,\mu_{eff} \,{\text{and}}\,\Gamma$, respectively.

The numerical analysis is performed using ANSYS Fluent^39^. Flow turbulence modeling is performed via the k-ω SST model, which is recognized for its numerical robustness. This model is adept at managing adverse pressure gradients and addressing scenarios involving flow separation. The model not only accurately evaluates the wall boundary layer and ensures stability and faster convergence of numerical simulation but also provides results that are better aligned with the experimental correlations^40–42^. Although it may slightly overestimate turbulence levels, its stable nature and reliability render it a preferred choice in such applications. At the inlet of the channel, the average flow velocity for air is specified on the basis of the required Reynolds number ($Re = 9000 - 24000$). Re is calculated based on the air density, viscosity, duct hydraulic diameter, and air inlet velocity (V) at an inlet temperature of 300 K. All the solid walls of the duct are treated as no-slip boundaries. A constant heat flux of $q=1000\, \text{W}/{\text{m}}^{2}$ is uniformly applied on the aluminum heating plate, forming the test section’s top surface^30,31^. The outflow condition, with atmospheric pressure of 1.01325 × 10^5^ Pa and zero gradients for all flow quantities, is specified at the duct outlet. All the remaining duct walls are considered adiabatic and impermeable. The average heat transfer coefficient of the air heater duct is evaluated on the basis of the heat flux applied to the heated plate as follows:

4 $$h = \frac{q}{{T_{w} - T_{a} }}$$

where $T_{w}$ is the average temperature of the heated plate and $T_{a} = \frac{{T_{i} + T_{o} }}{2}$ the mean temperature of the air in the test section, which is based on the inlet and outlet temperatures of the air. In the above expression, the surface area of the vortex generators is neglected because it is much smaller than the plate area. For the case of a constant heat flux heated plate, the arithmetic average is a valid method for estimating the mean fluid temperature, and it is also in conformity with the experimental method followed for developing the heat transfer correlations. The Nusselt number ($Nu$) is estimated via the equation below.

5 $$Nu = \frac{hD}{k}$$

where $k$ is the thermal conductivity of air and $D$ is the hydraulic diameter of the SAH^30,31^ .

The friction factor ($f$) is estimated on the basis of the pressure drop $\Delta p$ across the test section with $L = 800 \text{mm}$ via the equation below.

6 $$f = 2\left( {\frac{\Delta p}{L}} \right)\frac{D}{{\rho V^{2} }}$$

The Dittus–Boelter and Blasius equations are employed to validate the numerical results for configurations involving smooth ducts^5^. These correlations are widely recognized for their simplicity and applicability over a broad spectrum of Reynolds numbers within fully developed turbulent flow regimes. The Dittus–Boelter equation applicable for $Re > 10000$, $0.6 < Pr < 160$, and $L/D >10$ is given as follows:

7 $$Nu = 0.023\,\text{Re} ^{{0.8}}\, \text{Pr}^{{0.4}}$$

where $\Pr = {{\mu c_{p} } \mathord{\left/ {\vphantom {{\mu c_{p} } k}} \right. \kern-0pt} k}$ is the Prandtl number and $c_{p}$ the specific heat of the fluid^30,31^ . The Blasius equation for the friction factor of a smooth duct is given by

8 $$f = 0.316\,{\text{Re}}^{ - 0.25}$$

The increase in the Nusselt number results in an increase in heat convection, which is desirable in an SAH, but the simultaneous friction factor increase results in energy loss in terms of pumping power to overcome the additional pressure drop caused by the roughness elements. Therefore, thermal–hydraulic efficiency, the trade-off parameter given by the following relation, is used to assess the actual benefits of roughening the heating surface of the SAH. The effective thermal‒hydraulic performance (THP) is evaluated by considering the relative gain of heat transfer over the pressure drop penalty of the proposed design via the relation^5,13,34^.

9 $$THP = {{\left( {\frac{Nu}{{Nu_{S} }}} \right)} \mathord{\left/ {\vphantom {{\left( {\frac{Nu}{{Nu_{S} }}} \right)} {\left( {\frac{f}{{f_{S} }}} \right)^{{{1 \mathord{\left/ {\vphantom {1 3}} \right. \kern-0pt} 3}}} }}} \right. \kern-0pt} {\left( {\frac{f}{{f_{S} }}} \right)^{{{1 \mathord{\left/ {\vphantom {1 3}} \right. \kern-0pt} 3}}} }}$$

### Grid independence study and validation

**Fig. 2:**
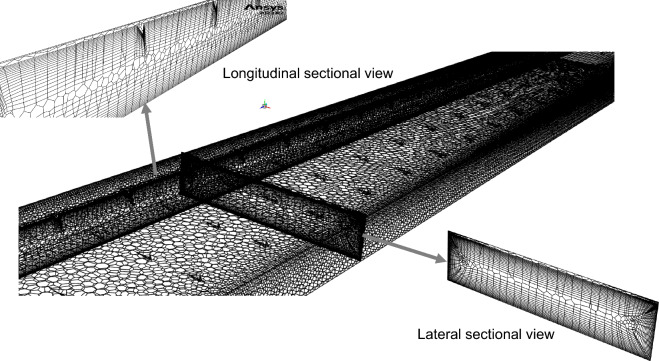
Computational mesh used in the numerical study.

The computational domain was discretized via ANSYS Fluent meshing. A high-quality mesh was created with polyhex-core meshing, which uses efficient hexahedral elements to reduce the cell count and optimize the simulation time. The flow domain next to the no-slip walls was meshed with an inflation layer consisting of 18 layers with growth rates and 1st layer heights of 1.1 and 0.2 mm, respectively. Figure 2 illustrates the mesh created through polyhex-core meshing for the SAH domain, including both longitudinal and lateral sectional views. The mesh quality is improved to achieve maximum skewness below 0.25 and maximum y + less than 10. This y + range effectively resolves the buffer layer by utilizing the k-ω formulation near the wall for detailed turbulence and transitioning to the k-ε model further away, striking a balance between accuracy and computational efficiency.

**Fig. 3:**
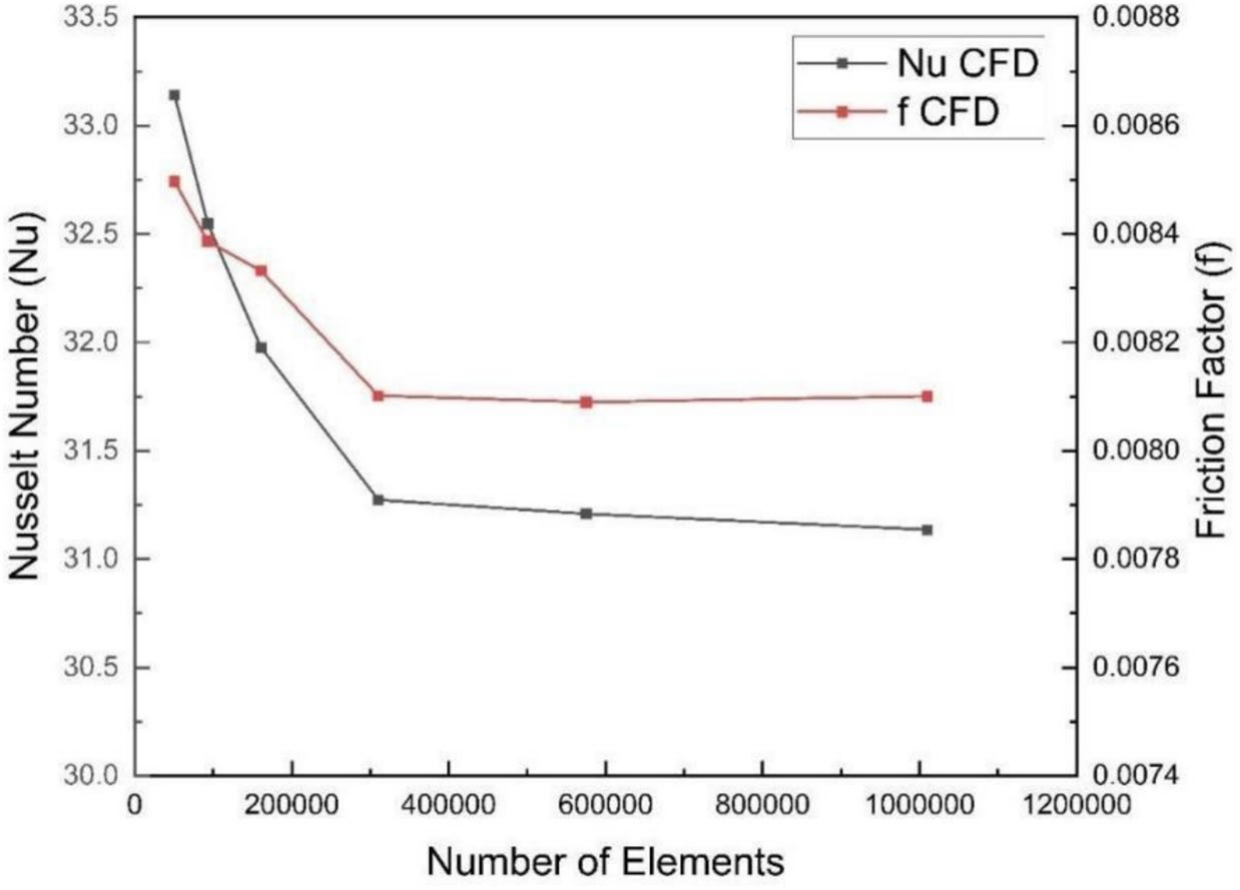
Nusselt number and friction factor values obtained for different mesh configurations at Re = 9000.

Numerical simulations were performed via various grid configurations. As shown in Fig. 3, after 310,433 elements were reached, the Nusselt number and friction factor results exhibited negligible changes, indicating grid independence. Thus, the grid parameters corresponding to this element count are used in further calculations.

**Fig. 4:**
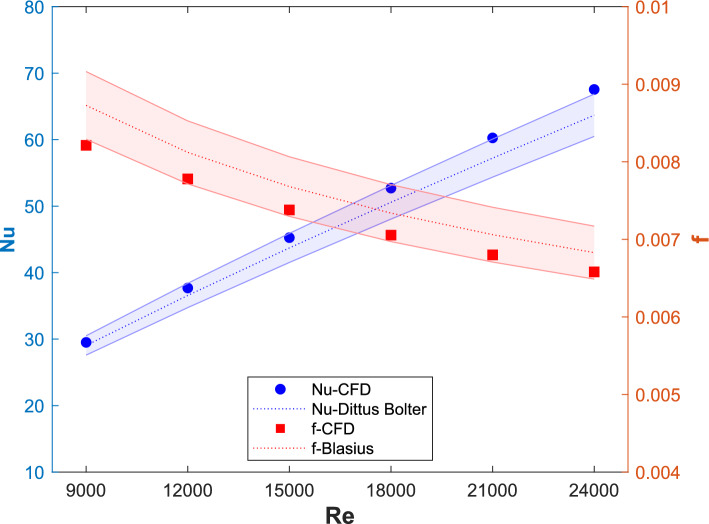
Validation of CFD model and comparison of the Nusselt number and friction factor values for smooth ducts.

The numerical results obtained from the *k*-ω SST turbulence model on a sufficiently fine mesh are then compared with the Dittus–Boelter and Blasius equations, as shown in Fig. 4. A 10 percent error band is shown for the equations and is compared with the CFD results. The minimum error observed for the Nusselt number is 1.6% at $Re=9000$ and steadily increases to 6.1% at $Re=24000$. For the friction factor, the error is 5.9% at $Re=9000$ and decreases to 3.6% at $Re=24000$. The numerical model shows an average deviation of 4% for the Nusselt number ($Nu$) and 4.2% for the friction factor compared with the correlation values, confirming its successful validation for further analysis.

## Results and discussions

To demonstrate the effectiveness of the proposed tetrahedron-shaped turbulator design, a performance comparison is conducted via a three-dimensional CFD model for both smooth and roughened heating surfaces. Tetrahedron turbulators are arranged in an inline pattern and connected to the heating plate. Initially, the effect of the longitudinal pitch of the turbulator is studied. The turbulators are set at a relative longitudinal roughness pitch ($P/D$) varying from 2.55 to 0.45, whereas the relative lateral roughness pitch ($p/D$) remains constant at 0.750. The number of turbulators increases as the value of the $P/D$ ratio decreases. That is, the $P/D$ configuration of 2.55 consists of 32 turbulators arranged in eight rows and four columns, whereas the $P/D$ configuration of 0.45 consists of 164 turbulators arranged in forty-one rows and four columns. The study also examines the effects of parameters such as the relative roughness height ($e/D$) and leading-edge angle ($\beta$). The roughness height ($e$) is measured orthogonally to the heated absorber plate, ranging from 3 to 9 mm in increments of 2 mm. Furthermore, the effect of the leading-edge angle ($\beta$) on thermo-hydraulic performance is analyzed by varying the angle from − 45° to 45°.

### Effect of the longitudinal pitch of the turbulators

The effect of changing the relative longitudinal pitch ($P/D$) between the turbulators on the thermal performance of the solar air heater is discussed here. The tested values for $P/D$ are 2.550, 1.116, 0.812, and 0.450, indicating an increase in the number of tabulators and a gradual reduction in the distance between them. The relative height of the turbulators ($e/D$) is maintained at 0.090, and the leading-edge angle ($\beta$) remains fixed at 0°.

**Fig. 5:**
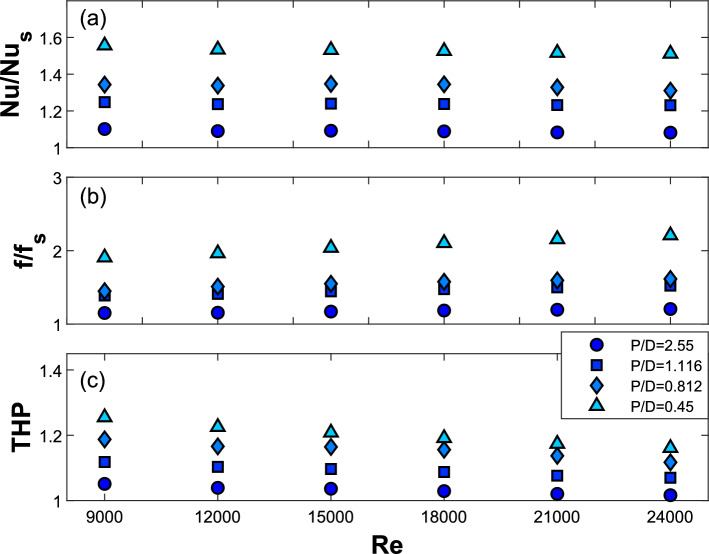
Variations in the (**a**) Nusselt number ratio, (**b**) friction factor ratio, and (**c**) THP parameter with the Reynolds number for different turbulator longitudinal pitches with e/D = 0.09, p/D = 0.75, β = 0°.

Figure 5a–c illustrate the variations in the Nusselt number ($Nu$) ratio, friction factor ratio, and THP parameter as a function of the Reynolds number ($Re$) for different relative longitudinal pitch values ($P/D$). These ratios represent the performance metrics of the SAH equipped with a turbulator in comparison to those measured without the turbulator. The THP parameter is estimated via Eq. 9. For a specific $P/D$ ratio, the Nusselt number ($Nu$) increases as the Reynolds number ($Re$) increases. However, as shown in Fig. 5a, the relative change in $Nu$ compared with that of a smooth duct remains relatively constant at higher Reynolds numbers. Additionally, for a given Reynolds number, the ratio of the Nusselt number is greater when the $P/D$ ratio is lower. In other words, as the $P/D$ ratio decreases, the number of turbulators on the heating surface increases. Adding turbulators to a solar air heater increases the Nusselt number by enhancing convective heat transfer through induced turbulence in the airflow. This turbulence disrupts the thermal boundary layer near the absorber surface, improving the mixing of hot and cold air and resulting in a greater temperature gradient at the wall. The consistent Nusselt number ratio observed across different Reynolds numbers can be better understood by analyzing the temperature distribution at the heated surface of the SAH.

**Fig. 6:**
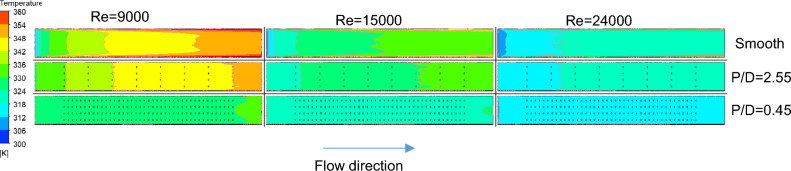
Temperature contours at the heated absorber plate for different longitudinal pitches and Re values.

Figure 6 shows the temperature contours measured along a plane parallel to the heated absorber plate at a depth of 1 mm below it. Solar air heaters without a turbulator and those with $P/D$ ratios of 2.55 and 0.45 are compared for three Reynolds numbers. For the SAH with no turbulator, at $Re=9000$, the temperature at the given plane gradually increases from the entrance to the exit of the test section. The impact of the no-slip condition of the sidewalls is noticeable at lower Reynolds numbers as the flow moves toward the exit. As the flow velocity corresponding to the Reynolds number increases, heat transfer improves, reducing the temperature in the chamber. In the solar air heater with a $P/D$ ratio of 2.55, the temperatures are lower than those without turbulators, and this effect is further enhanced when the $P/D$ ratio is reduced to 0.45. At a $P/D$ ratio of 0.45, the temperature distribution exhibits a nearly uniform profile across the entire surface for all three Reynolds numbers, albeit with variations in the specific temperature values observed. This indicates that the Nusselt ratio will remain relatively constant despite variations in flow conditions, as illustrated in Fig. 5a. As the $P/D$ value decreases, increasing the number of turbulators helps suppress the redevelopment of the laminar sublayer on the heating surface and enhances turbulence. This results in improved heat convection from the surface.

**Fig. 7:**
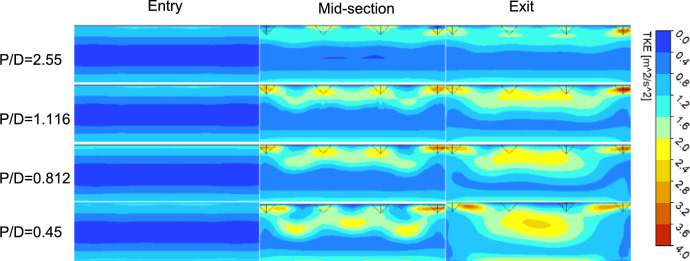
Turbulent kinetic energy (TKE) contours at the heating surface for three different P/D values at Re = 24,000.

The increase in turbulence due to a greater number of turbulators is illustrated by the turbulence intensity contours of the SAH, as presented in Fig. 7. The turbulent kinetic energy of the fluid is shown in the cross-sections at the entry, middle, and exit of the test section, corresponding to different $P/D$ values for a given Reynolds number of 24,000. The TKE is calculated as

10 $$TKE = \frac{1}{2}\left( {\overline{{u^{{\prime 2}} }} + \overline{{v^{{\prime 2}} }} + \overline{{w^{{\prime 2}} }} } \right)$$

where $u^{\prime } ,v^{\prime } ,w^{\prime }$= velocity fluctuations in the $x$, $y$, and $z$ directions and $\overline{{u^{{\prime 2}} }} ,\overline{{v^{{\prime 2}} }} ,~\overline{{w^{{\prime 2}} }}$ time-averaged squares of the velocity fluctuations^43^. For a given $P/D$ ratio, the turbulent kinetic energy increases as the flow moves from the entry to the exit of the test section. At a $P/D$ ratio of 2.55, elevated levels of turbulent kinetic energy are detected predominantly in the uppermost layers of air in the SAH, which is in proximity to the turbulators. However, as the $P/D$ values decrease (e.g., $P/D=0.45$), the turbulent kinetic energy begins to distribute toward the lower bulk region in the SAH chamber. This finding indicates that increasing the number of turbulence-inducing elements facilitated flow detachment and enhanced mixing within the SAH chamber. Notably, even though the turbulators increase turbulence and improve heat transfer, the increased number of rough elements also leads to greater energy loss due to friction as the fluid flows over the roughened surface.

**Fig. 8:**
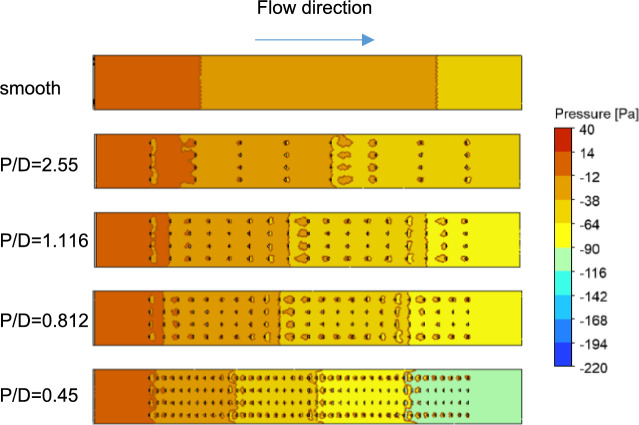
Pressure contours at the heating surface for three different P/D values at Re = 24,000.

The friction factor,$f$, decreases with increasing Reynolds number at a constant $P/D$. However, the friction factor gradually increases when the longitudinal pitch changes from $P/D=2.550 \,\text{to}\, 0.45$. This increase in $f$ can be attributed to the greater number of turbulators in the test section as the $P/D$ ratio decreases, leading to an increased pressure drop in the system. The variation in the friction factor ratio at different Reynolds numbers for various $P/D$ values is shown in Fig. 5b. The friction factor ratio is lower at low $Re$ values and increases with $Re$. For a given Reynolds number, the normalized friction factor is elevated at lower $P/D$ ratios. This phenomenon can be attributed to an increase in the number of turbulators, resulting in a corresponding increase in the pressure drop. The friction factor for the smooth duct decreases more rapidly with increasing Reynolds number than in the roughened duct with tetrahedron turbulators, particularly at lower $P/D$ ratios with more turbulators. Consequently, while $f$ decreases for both ducts, the ratio $f/fs$ can increase. At higher $P/D$ ratios, the $f-Re$ trends for both ducts align, resulting in a nearly constant $f/fs$. The pressure contours along the flow direction in the SAH both with and without turbulators, for a Reynolds number of 24,000, are illustrated in Fig. 8. In a smooth channel without any turbulators, the pressure drop across the chamber is lower than that observed in the SAH with turbulators.

As both the heat transfer rate and pressure drop increase with the addition of turbulators, evaluating the THP parameter will provide insight into the overall performance of the solar air heater with these turbulators. Figure 5c shows the variation in THP with respect to $Re$ for different $P/D$ ratios. The THP decreases with increasing $Re$ for all $P/D$ values. At a lower Reynolds number ($Re = 9000$), the THP values range from 1.05 to 1.25, indicating improved thermal performance in relation to the penalties associated with pumping losses, whereas at higher Reynolds numbers ($Re = 24000$), the THP values vary between 1.01 and 1.16. Additionally, a lower $P/D$ ratio is associated with higher THP values; specifically, as the number of turbulators increases, there is a significant increase in heat transfer despite an increase in the pressure drop. For a $P/D$ ratio of 0.812 with a total of 92 turbulators, the THP values range from 1.11–1.18.

The THP values show only a 3% improvement when the $P/D$ ratio is reduced from 0.812 to 0.45, despite nearly doubling the number of turbulators from 92 to 164. Given the minimal performance gain and the increased computational cost for the $P/D = 0.45$ configuration, this study will continue with $P/D = 0.812$. This choice enables a more efficient and focused investigation of the effects of turbulator height and leading-edge angle.

### Effect of the turbulator height

To analyze the effect of the turbulator height, the relative roughness height ($e/D$) is varied, with values set at 0.090, 0.150, 0.210, and 0.270, corresponding to turbulator heights of 3, 5, 7, and 9 mm, respectively.

**Fig. 9:**
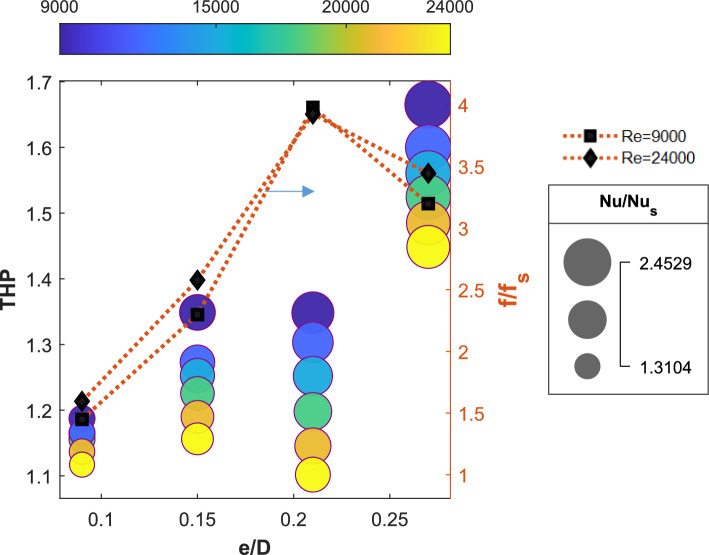
Variations in the Nusselt number ratio, friction factor ratio, and thermo-hydraulic performance parameter with the Reynolds number for various turbulator heights with P/D = 0.812, p/D = 0.75, β = 0⁰.

The observation made by changing the turbulator height is consolidated as a bubble graph in Fig. 9. The size of the bubble represents the Nusselt number ratio, and the color of the bubble represents the Reynolds number. The values of THP are plotted toward the left axis, whereas the friction factor ratios for $Re=9000$ and $Re=24000$ are plotted toward the right axis. The turbulator heights are plotted on the x-axis. The values of the Nusselt number ratio are observed to be elevated at lower Reynolds numbers. This phenomenon is evidenced by a decrease in bubble size with an increase in $Re$ at a specified turbulator height. Furthermore, a higher Nusselt number ratio is noted at greater turbulator heights. For a Reynolds number of 24,000, the $Nu$ ratio for the SAH with turbulators of 9 mm height is 2.2, whereas this value decreases to 1.3 when the turbulator height is reduced to 3 mm. The relative friction factor ratio is denoted by the dotted lines in Fig. 9 for two $Re$ values. The friction factor ratio increases with increased turbulator height until 7 mm; however, at a 9 mm turbulator height, the ratio decreases. This can be explained on the basis of the pressure contours on the downstream side of the turbulators at different heights.

**Fig. 10:**
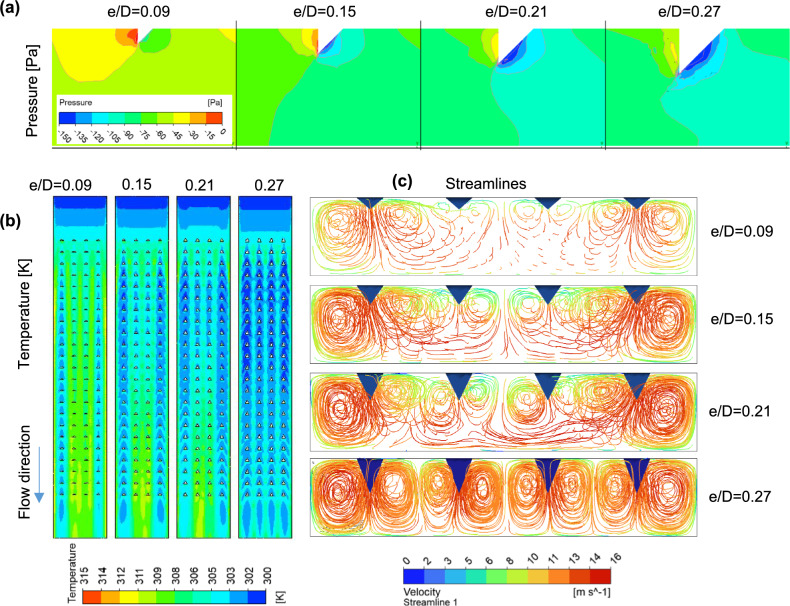
(**a**) Pressure profile on the mid-section turbulator, (**b**) temperature profile at the heated absorber plate, and (**c**) streamline observed from the exit for the SAH for various e/D ratios with p/D = 0.75, P/D = 0.812, and β = 0 at Re = 24,000.

Figure 10a shows the pressure contour plots for $e/D$ values ranging from 0.09 to 0.27. The turbulator located at the middle portion of the test section in the first column of the array is used for the analysis. The contour maps indicate that the pressure difference between the upstream and downstream sides of the turbulator increases with increasing height up to $e/D=0.21$. Beyond the $e/D$ value of 0.21, the pressure profile shows a reduction in the pressure drop across the turbulator, resulting in a lower friction factor ratio. The anomalous reduction in the friction factor beyond $e/D=0.21$ may be due to the turbulators generating coherent swirling vortices, leading to a more organized flow, as seen in Fig. 10c for $e/D=0.27$. Stable recirculation zones downstream of the turbulators shield the subsequent turbulator walls from direct flow impingement, reducing form drag compared to chaotic separations. Figure 10b shows the temperature profile on the plane close to the heated absorber plate. Efficient heat transfer from the heated absorber plate to the air results in a lower surface temperature. The temperature at the exit section is greater when the $e/D$ ratio is small, and it decreases as the height of the turbulators increases. This reduction in temperature is caused by the mixing of the fluid facilitated by the turbulators. This can be understood by visualizing the streamlines within the SAH. A comprehensible visual view of mixing can be obtained by viewing through the exit port of the SAH. Figure 10c shows the streamlines as they appear at the outlet of the SAH when viewed normally for varying values of $e/D$. While plotting, the number of streamlines is kept the same ($n= 500$) for all the cases for consistency in comparison. Mixing within the SAH clearly increases with increasing turbulator height. For an $e/D$ value of 0.09, not much mixing is caused by the turbulators, especially in the middle columns. As a result, the air exits at a lower temperature, resulting in a higher temperature zone at the exit portion of the heated absorber plate. Notably, the sidewalls also contribute to mixing. The stagnant areas at the corners where the adjacent walls intersect create higher temperature zones, which can be seen at the right and left sides of the temperature profile at the heated absorber plate (Fig. 10b). The increased mixing resulting from taller turbulators accounts for the higher Nusselt ($Nu$) ratios observed in Fig. 9. Since the $Nu$ ratio is higher and the friction ratio is low, the thermo-hydraulic performance parameter will be greater when the height of the turbulator is increased beyond 7 mm. This is observable from the location of the bubbles depicting the THP values in Fig. 9. The THP values for turbulator heights of 5 mm and 7 mm are of the same order. This occurred because even though $Nu$ increased with increasing turbulator height, the pressure drop also increased simultaneously. A THP value of 1.66 is observed for the SAH for a turbulator height of 9 mm for a $Re$ value of 9000. For all the cases discussed above, the leading-edge angle ($\beta$) is kept at a value of zero degrees. As the maximum THP is obtained for a turbulator height of 9 mm, further analysis to investigate the effect of the leading-edge angle is performed at this height.

### Effect of the turbulator leading edge angle ($\beta$*)*

**Fig. 11:**
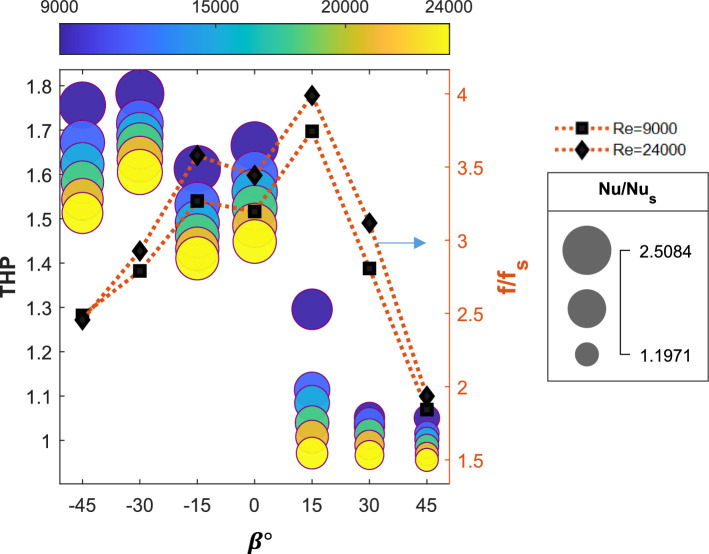
Variations in the Nusselt number ratio, friction factor ratio, and thermo-hydraulic performance parameter with the Reynolds number for various turbulator leading-edge angles with p/D = 0.75, P/D = 0.812, and e/D = 0.27.

**Fig. 12:**
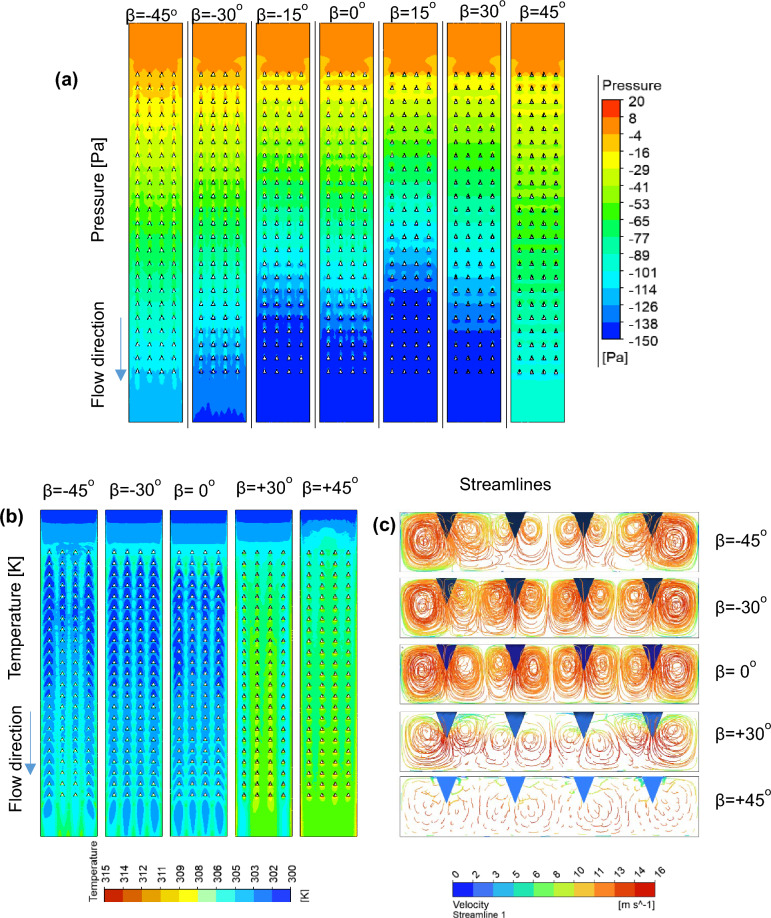
(**a**) Pressure profile at the heated absorber plate, (**b**) temperature profile at the heated absorber plate, and (**c**) streamline observed from the exit for the SAH for various leading-edge angles with p/D = 0.75, P/D = 0.812, and e/D = 0.27 at Re = 24,000.

The influence of the leading-edge angle, ranging from − 45° to + 45°, is analyzed. The negative leading-edge angle refers to the swaying of the tetrahedral turbulator oriented against the flow, whereas the positive leading-edge angle denotes the swaying aligned with the flow direction. Similar to Fig. 9, the impact of the leading-edge angle on the Nu ratio and THP is illustrated as a bubble graph in Fig. 11, with the size, location, and color of each bubble representing the $Nu$ ratio, THP, and $Re$, respectively. The friction factor ratios are shown on the right side of the y-axis of the plot for two different Reynolds number values. The friction factor ratios increase as the leading-edge angle varies from − 45° to 15° and further decrease with increasing angle. This is caused mainly by the reduction in the frontal area projected by the turbulator against the flow. The observation is more evident from the pressure profile obtained at the mid-section of the SAH, as shown in Fig. 12a. Here, pressure profiles for varying leading edge angles are plotted at $Re=24000$. The pressure drop between the inlet and exit of the SAH is the lowest when $\beta$ is + 45°, as the turbulator’s swaying angle aligns with the flow direction, resulting in minimal disturbance to the flow.

Figure 11 shows that the $Nu$ ratio values are greater at negative leading-edge angles than at positive leading-edge angles. Additionally, at any given leading-edge angle, the variation in the $Nu$ ratio with the Reynolds number is more pronounced at negative values of $\beta$. The higher $Nu$ ratio values for negative $\beta$ angles are evident from the temperature profile plot at the heated absorber plate given in Fig. 12). Turbulators with a leading-edge angle of + 45° show limited mixing, resulting in a high temperature at the heated absorber plate even in the entrance zone of the SAH. In contrast, the temperature contours for a leading-edge angle of $\beta =-30^\circ$ are similar to those observed for $\beta =0^\circ$. The highly mixed nature of air at $\beta =-30^\circ$ and the nonmixing nature at $\beta =+45^\circ$ are evident from the streamline plot obtained at the exit port of the SAH, as depicted in Fig. 12c. The mixing patterns observed in the streamlines at β =  − 30° and $\beta =0^\circ$ exhibit notable similarity, which accounts for the comparable $Nu$ ratios recorded for these conditions.

### Comparison of the tetrahedral turbulator with others in the literature

Table 2 shows a comparison of the THP values reported in the literature with those reported in the present study. Our results are compared with those that are very close to the tetrahedron shape, such as the winglet and conical turbulators. The table shows that the proposed tetrahedron-shaped turbulators perform comparably well with most of the designs in terms of THP. Notably, higher THP values in the literature were reported at lower $Re$ values (at ~ 4000), whereas our study started with a minimum $Re$ value of 9000. At lower Reynolds numbers, higher THP is achievable with tetrahedron-shaped turbulators. However, these lower flow rates are undesirable as they tend to increase the surface temperature of the heated absorber plate beyond safe limits. The current study focused on the inline arrangement of turbulators. In the future, we will extend the analysis for the staggered arrangement.

**Table 2:** Comparison of the THP for different turbulator designs.

SI. No	Turbulator geometry	Maximum THP	Parameter set corresponding to maximum THP
1	Trapezoidal winglet and wavy groove^44^	2.76	$Re: 4780$ 45° angle of attack
2	Perforated winglet^34^	2.01–1.78	$Re: 4100-25500$ $d=5 \text{mm}$ 30° angle of attack
3	V down baffle blocks with racetrack-shaped holes^26^	1.43	$Re: 13000$ $D=53.65 \text{mm}$
4	Conical Protrusion Ribs^27^	1.346	$Re: 4000$ $e/D=0.044$,$p/e=10$
5	Tetrahedron-shaped turbulators [Present study]	1.78–1.61	$Re: 9000 - 24000$ $e/D=0.27$, $P/D=0.812$ $p/D=0.75$,$\beta =-30^\circ$

It may also be noted that the presence of turbulators affects flow dynamics in two main, interconnected ways: it increases turbulent mixing, which enhances convective heat transfer, and it creates localized stagnation or recirculation zones that prolong the air’s residence time. This combination improves thermal performance; however, it may also result in higher pressure penalties. Understanding this interaction is essential for optimizing turbulator design to achieve maximum thermo-hydraulic efficiency. Therefore, future studies could explore transient simulations with particle tracking to better understand the effect of flow residence time.

## Conclusions

A CFD analysis is conducted to assess the heat transfer performance of a solar air heater (SAH) equipped with tetrahedron-shaped turbulators. The study measures the thermo-hydraulic performance (THP) parameter for Reynolds numbers ranging from 9,000 to 24,000. The results indicate that the turbulators enhance heat transfer by increasing turbulence, which improves the effective THP, although this comes with a higher pressure drop. The salient conclusions from the study are as follows:

- The incorporation of turbulators in a solar air heater significantly enhances the Nusselt number by improving convective heat transfer through the induction of turbulence in the airflow. For a given Reynolds number, the Nusselt number ratio between a SAH with tetrahedron-shaped turbulators and one without increases as the $P/D$ ratio decreases.
- Both the Nusselt number and the friction factor increased with increasing height of the turbulators up to 7 mm ($e/D=0.21$), and beyond this height, the friction factor slightly reduced, yielding the highest THP values of 1.66 for the turbulator height of 9 mm ($e/D=0.27$) at $Re=9000$.
- A negative leading-edge angle enhances heat transfer by improving mixing, whereas a positive angle results in decreased mixing and lower THP values. Notably, the leading-edge angle of $\beta =-30^\circ$ demonstrates superior effectiveness compared to 0°.
- On the basis of the numerical study, the tetrahedron-shaped turbulator with a relative longitudinal pitch ($P/D$) = 0.812, relative turbulator height ($e/D$) = 0.270, relative lateral pitch ($p/D$) = 0.750, leading edge angle $\beta =-30^\circ$, and angle of attack $\alpha =30^\circ$ yielded the highest THP value of 1.78.

## Data Availability

The datasets generated and/or analysed during the current study are available in the figshare repository at https://figshare.com/s/2964b9399f745f3d505a. The additional numerical simulation datasets used and/or analysed during the current study are available from the corresponding author on reasonable request.

## List of symbols

$c_{p}$: Specific heat of fluid (J/(kg K))
*D*: Hydraulic diameter (m)
*e*: Height of the turbulator (m)
*e/D*: Relative roughness height
*H*: Duct height (m)
*f*: Friction factor
*h*: Convective heat transfer coefficient (W/(m^2^ K))
*l*: Base length of the turbulator (m)
*L*: Test section length (m)
*k*: Fluid thermal conductivity (W/(m K))
*Nu*: Nusselt number
*P/D*: Relative longitudinal pitch
*p/D*: Relative lateral pitch
*Pr*: Prandtl number
*q*: Heat flux (W/m^2^)
*Re*: Reynold number
*T*: Temperature (K)
*V*: Velocity (m/s)
*W*: Duct width (m)
CFD: Computational fluid dynamics
SAH: Solar air heater
THP: Thermo-hydraulic performance
TKE: Turbulent kinetic energy

## Greek symbols

*α*: Angle of attack
*β*: Leading edge angle
*Δ*: Difference
$\rho$: Density of fluid (kg/m^3^)

## Subscripts

*a*: Average
*i*: Inlet
*o*: Outlet
*s*: Smooth
*w*: Wall (absorber plate)
